# Antibacterial and plant growth-promoting properties of novel Fe_3_O_4_/Cu/CuO magnetic nanoparticles

**DOI:** 10.1039/d2ra03114k

**Published:** 2022-07-07

**Authors:** Zhifeng Liu, Shaobo Guo, Xun Fang, Xianzhao Shao, Zuoping Zhao

**Affiliations:** Shaanxi Key Laboratory of Catalysis, School of Chemical & Environment Science, Shaanxi University of Technology Hanzhong Shaanxi 723001 China lzhifeng2005@126.com +86-0916-2641660 +86-0916-2641660; State Key Laboratory of Qinba Bio-Resource and Ecological Environment, Shaanxi University of Technology Hanzhong Shaanxi 723001 China

## Abstract

In this work, an Fe_3_O_4_/Cu/CuO (FC) antibacterial nano-agent was synthesized in a “one-pot” approach using copper sulfate and ferric chloride as raw materials, and it was studied using TEM, XRD, XPS, UV-vis, and VSM methods. The antibacterial activity and mechanism of FC were studied, using a commercially available Bordeaux mixture as a control. The effects of an FC on mung bean development and its toxicity to human mammary epithelial cells were also investigated. The results revealed that FC could break the cell walls of *E. coli* and *S. aureus*, quadrupling the antibacterial activity of the Bordeaux combination. Furthermore, it was shown that FC might improve the germination, root development, and chlorophyll content of mung bean seeds while being 1/8 as hazardous to human mammary epithelial cells as the Bordeaux combination. The as-prepared FC can replace the Bordeaux combination in the management of agroforestry pathogens.

## Abstract 2

In this work, an Fe_3_O_4_/Cu/CuO (FC) antibacterial nano-agent was synthesized in a “one-pot” approach using copper sulfate and ferric chloride as raw materials, and it was studied using TEM, XRD, XPS, UV-vis, and VSM methods.

## Graphical abstract

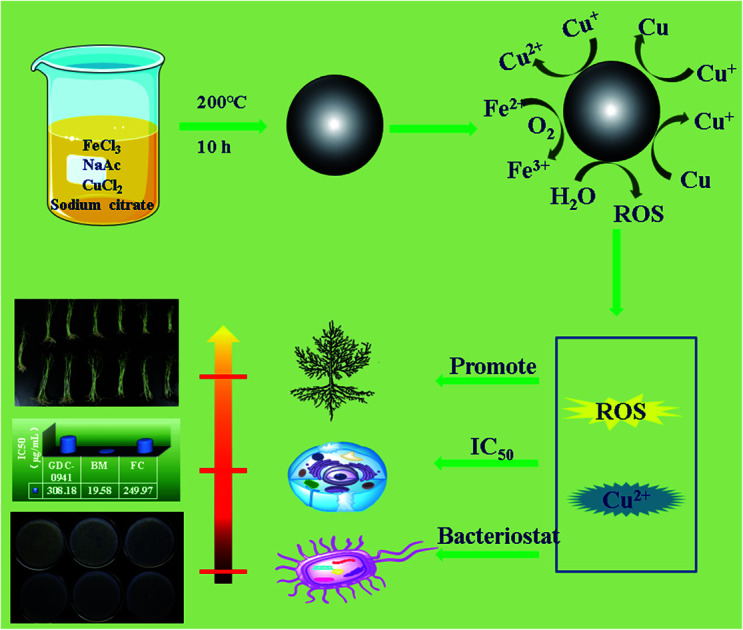

## 1. Introduction

Copper ions, as trace elements in plants, are required for various physiological functions, including photosynthesis, cellular respiration, antioxidant defenses, and hormone signal transduction.^1–6^ Due to its exceptional biocompatibility, it has a wide range of applications. According to their biological value, copper ions can not only boost plant development but also have antibacterial properties due to their toxicity. On the other hand, being a heavy metal, copper may be harmful to plants, bacteria and viruses to a certain extent.^3,4,6–8^

Cu^2+^ is the primary component of commercial inorganic antibacterial agents such as Bordeaux mixture, green depot, and double spirit, which are extensively used in agriculture, forestry, and animal husbandry as a disposable dosage for disease prevention and control.^2,3,5^ Cu^2+^ ions can adsorb on the surface of bacteria, destroying the potential difference between the surface membrane and the bacterial cell walls.^9,10^ When paired with a sulfur iron cluster in the cell membrane, trace levels of Cu^2+^ can alter the secondary structure of the protein in the ion channel, resulting in the rupture of bacterial cell walls.^9,11^ Proper amounts of Cu^+^ drifting through the cytoplasm (cell walls contain functional reductase, which can reduce Cu^2+^ to Cu^+^ and transport it into the bacterial interior *via* the copper ion channel protein) can combine with the cytoplasm to form oxides such as oxygen free radicals, peroxides, and oxygen ions, which can damage critical organelles, such as mitochondria,^12,13^ nucleic acid and plasmids, ultimately killing the bacteria. Thus, when the concentration of Cu ion is greater than the minimum inhibitory concentration for bacteria but less than the maximum inhibitory concentration for plants, it can be used to prevent and control agricultural pathogens due to its antibacterial properties and growth-promoting properties.^11,14^

However, when the environment changes and the usage of Cu ion-based antibacterial treatments increases, resistance genes are triggered in bacterial plasmids, resulting in increased production of the bacterial “efflux pump” system and solid Cu proteins.^10,12^ This results in drug-resistant bacteria and even super-bacteria, eventually diminishing the use of copper ion-based antibacterial treatments.

Copper nanoparticles (NCs, Table 1) exhibit significant surface plasmas. After absorbing a given amount of energy, electrons on its surface often escape and interact with the surrounding medium to form reactive oxygen species (ROS, Table 1), which can cause irreparable damage to Gram-positive and Gram-negative bacteria, viruses, fungi and mycoplasma.^13–15^ Meanwhile, NCs create Cu^2+^ when they release electrons, which in conjunction with ROS, confers on NCs significant antibacterial characteristics.

However, NCs are difficult to prepare and recover, and it is unstable when exposed to the environment. Wang *et al.*^16^ demonstrated that Fe^2+^ included in Fe_3_O_4_@Cu@Cu_2_O composites accelerated electron transport and generated reactive oxygen species (ROS), endowing the composite with increased stability and catalytic activity. Liu *et al.*^17^ produced Fe_3_O_4_@Cu@CuO composites to investigate their electrochemical characteristics. They discovered that Fe_3_O_4_@Cu@CuO had a high electron transfer efficiency and could be recycled more than 550 times.

Fe and Cu ions can work synergistically as trace elements in plants to promote plant metabolism and stem and leaf development. Given the excellent biocompatibility of Fe^2+^, Fe^3+^, Cu^0^ and Cu^2+^, ROS created by electron migration can potentially not only oxidize the seed coat, but boost aerobic respiration. To increase seed germination efficiency, the ions can also cause irreparable harm to bacteria when combined with Cu^2+^.

As a result, this work adopted the “one-pot approach” to create nanosphere FC composite material in our investigation. Gram-positive *S. aureus* and Gram-negative *E. coli* were utilized as model bacteria. A commercially available Bordeaux mixture was used as a control to investigate the material's antibacterial capabilities and mechanism of action. Meanwhile, the impacts on human mammary epithelial cells and mung bean germination were studied, focusing on its root activity and changes in chlorophyll content during the process.

## 2. Experimental procedure

### 2.1 Materials

FeCl_3_, NaCl, ethylene glycol and NaAc were purchased from Tianjin Damao Chemical Reagent Factory. Copper(ii) chloride was from Tianjin Zhiyuan Chemical Reagent Co. Ltd Bordeaux mixture was obtained from the U.S. Cerexagri Inc., yeast extract, tryptone and agar from Shanghai Zhanyun Chemical Co. Ltd *Staphylococcus aureus* and *Escherichia coli* were provided by Shaanxi Institute of Edible Fungi.

The microstructure of the sample material (FC nanosphere) in our study was visualized using transmission electron microscopy (TEM, Tecnai, FEI). Crystallinity was investigated using the D8 ADVANCE X-ray powder diffractometer (BRUKER, Germany), at target CuKα (*λ* = 0.154 nm), voltage 40 kV, current 35 mA, and scanning speed 2*θ* = 7°. Ultraviolet-visible diffuse reflection absorption spectroscopy (Cray 100, Shimadzu) at a scanning rate of 200 nm min^−1^ and a scanning range of 200–800 nm was utilized to test the molecular structure of the material. The magnetization curves of the FC were recorded using a model 7407 vibrating sample magnetometer (Lake Shore Cryotronics Inc., USA). The test temperature was set at 20 °C, and the magnetic field range was adjusted between −15 000 and 15 000 Oe (1 Oe = 10^−4^ T).

**Table 1:** The abbreviation list of the manuscript

Species	Abbreviation	Species	Abbreviation
Fe_3_O_4_/Cu/CuO	FC	Transmission electron microscope	TEM
Copper nanoparticles	NCs	X-ray photoelectron spectroscopy	XPS
Reactive oxygen species	ROS	Vibrating sample magnetometer	VSM
Bordeaux mixture	BM	Magnetic saturation	MS
Propidium iodide	PI	Ultraviolet visible	UV-vis
Luria-Bertani	LB	Human mammary epithelial cells	McF-7
Inhibitory concentration	IC_50_	Triphenyl tetrazolium chloride	TTC
X-ray diffraction	XRD		

### 2.2 Sample preparation

FC was synthesized using the hydrothermal method.^18^ In summary, 2.4 mM ferric chloride and 1.2 mM copper chloride were mixed and added to 20 mL of ethylene glycol, and the mixture was further ultrasonicated for 0.5 h, followed by the addition of NaAc (1.2 g) and sodium citrate (0.2 g) to the above solution for another ultrasonication treatment for 1 h. The mixture was then transferred to the Teflon vessel in a 25 mL stainless steel autoclave and reacted at 200 °C for 10 h. The solid black product was obtained and washed several times with ultrapure water and ethanol, and then dried for 12 h in a vacuum drying oven at 60 °C.

### 2.3 Monitoring ROS generation

Ascorbic acid has a characteristic UV absorption peak at 266 nm and can be oxidized by ROS to nonabsorbent deoxyascorbic acid.^19^ The generation of ROS is monitored in our work using this method. According to the specific procedure, 10 mL phosphoric acid buffer (PBS, pH = 7.4) was mixed with FC (16 μg mL^−1^), and then ascorbic acid (60 μM) was added. Subsequently, the mixture was grown at 37 °C for 20 minutes and removed nanomaterial by magnetic separation. Then, changes in ascorbic acid were monitored by UV-vis.

### 2.4 Stability test

To determine the stability of the FC in the medium, a PH = 7.4 phosphate buffer was used as a simulated biological solution, and UV-vis was used to detect the degree of change of the material over 48 h. To achieve this goal, 2 mg of FC was added to 50 mL of phosphate buffer and the change curves of the material were examined at 0 h, 6 h, 12 h, 24 h, and 48 h, respectively.

### 2.5 Bacterial inhibition test

*S. aureus* and *E. coli* were selected as model bacteria. LB medium, ultra-pure water, normal saline, phosphoric acid buffer and other biological materials used were sterilized in an autoclave at 121 °C for 20 min. Bacteria were cultured in the incubator at 37 °C, activated overnight before the test, and then diluted with sterilized distilled water into different concentrations. All operations were performed in a sterile environment, the experiment was performed six times and the data were averaged.

#### 2.5.1 Filter paper diffusion method

The activated bacterial suspension was further diluted to 5 × 10^7^ CFU mL^−1^ with sterile saline, and 50 μL bacterial suspension was evenly spread on the LB solid medium that had been sterilized. Sterile filter paper (7 mm in diameter) with 3 μL samples (containing different concentrations of nanocomposites in phosphate buffer pH = 7.2) was placed on the medium mentioned above.^20^ The experiment was repeated six times, and the results were observed after a 12 hours culture at 37 °C.

#### 2.5.2 Colony counting method

The sample nanomaterial was added to 5 × 10^5^ CFU mL^−1^ bacterial suspension until it reached a material concentration of 100 μg mL^−1^. It was then stirred for 5, 10, 20 and 40 minutes respectively. 10 μL of supernatant was obtained by magnetic separation and then evenly coated on the solid LB medium.^21^ The above experiment was repeated six times, and the results were observed after a 12 hours culture. Antibacterial efficiency (*n*) can be calculated from the following eqn (1).1
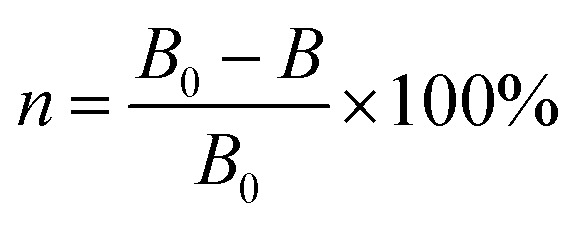
where *n* stands for antibacterial efficiency; *B*_0_ is the number of bacterial colonies in contrast, and *B* is the number of colonies after applying different materials.

#### 2.5.3 Monitoring of bacterial growth curve

The sample material and the liquid LB medium containing the bacteria were mixed into a 5 mL solution with a bacterial concentration of 5 × 10^7^ CFU and a material concentration of 100 μg mL^−1^. The solution was then added to the mixing and reaction C80 micro-colorimeter to be monitored at 37 °C for the growth of the bacteria and its heat release intensity.

#### 2.5.4 Bacteria PI staining experiment

Added 50 μL of propidium iodide (PI) (50 μg mL^−1^) to the above-mentioned solution (1 mL) and stirred in the dark for 15 min.^22^ The solution was then washed with phosphoric acid buffer 3 times in a centrifuge at 13 000 r per min. Following that, we observed the bacterial damage under an inverted fluorescence microscope.

### 2.6 Experiment of toxicology

Human mammary epithelial cells (McF-7) at a standard concentration of 2000 cells per well were added to 96-well plates (10% standard FETAL bovine serum HDMEM medium) for overnight adhesion culture. Then, the HDMEM medium was removed and 100 μL of medium containing different concentrations of sample material was added. After 3 days of culture at 37 °C with the 5% CO_2_ level and 95% humidity level, 25 μL MTT solution (5 mg mL^−1^ in PBS) was added and cultured for 2 hours. Afterward, the supernatant was removed and 100 μL DMSO was added to dissolve the Formazan crystals.^23^ The medium was then sealed for overnight culture. A spectrophotometer was used to monitor absorption peaks to assess the toxicity (*W*) of the material to cells. The experiment was repeated four times and the results were averaged. The equation of calculation is as followed eqn (2).

2 *W* = OD (experimental group)/OD (control group) × 100%

### 2.7 Plant growth experiment

#### 2.7.1 Seed germination experiment

Plumpy mung bean seeds were immersed in 10% (v/v) NaClO_4_ for sterilization, cleaned with distilled water several times, and then soaked in distilled water at 4 °C overnight. Soaked seeds were evenly spread in a Petri dish, with 20 seeds in each.^24^ This operation was repeated six times. Ultrapure water and a solution with a given concentration of the FC were added to the Petri dish. Germination was observed in the environmental incubator at 25 °C for 36 hours. The experiment was performed with 6 replications and the results were averaged.

#### 2.7.2 Root activity and chlorophyll content measuring experiment

Raw mung bean seeds were added to a nutrient solution and cultured at 25 °C until germination.^25,26^ Subsequently, the seedlings were transferred to a nutrient solution with a concentration gradient of sample material. The seedlings were then placed in an artificial climate incubator at 25 °C, with humidity at 60–70%, illumination time of 16 h day^−1^, and illumination intensity of 150 μmol s^−1^ m^−2^. Root growth activity and chlorophyll content were measured after 7 days of treatment. The operation was performed with six replications and the results were averaged.

##### (1) Root activity measurement

The triphenyl tetrazolium chloride (TTC) method was used to monitor the root growth activities of mung bean sprouts. 5 mL of TTC solution (4 g L^−1^) and 5 mL of phosphoric acid buffer (0.07 mol L^−1^) were added successively to the mung bean sprout roots (0.5 g) and they were mixed well.

After, the seeds were incubated in the dark at 37 °C for 2 h. Then 2 mL of H_2_SO_4_ (1 M) was added to the roots to stop the reaction. The sprouted roots were then removed and wiped clean. Following that, they were placed in a graduated test tube and 10 mL of methanol was added to the test tube. Wait until the root tip turns completely white at 25 °C. Then, the supernatant was taken and the amount of tetrazolium reduction was monitored spectrophotometrically.

##### (2) Chlorophyll content measurement

The leaves of 0.15 g of mung bean sprouts were taken and ground into a powder with liquid nitrogen for 20 min. Then the powder was soaked in a mixture of 20 mL of acetone and ethanol (v/v = 1 : 1) for 24 h. After that, the supernatant was taken and its chlorophyll content was measured by the spectrophotometer.

## 3. Results and discussion

### 3.1 Morphology and crystal type

**Fig. 1:**
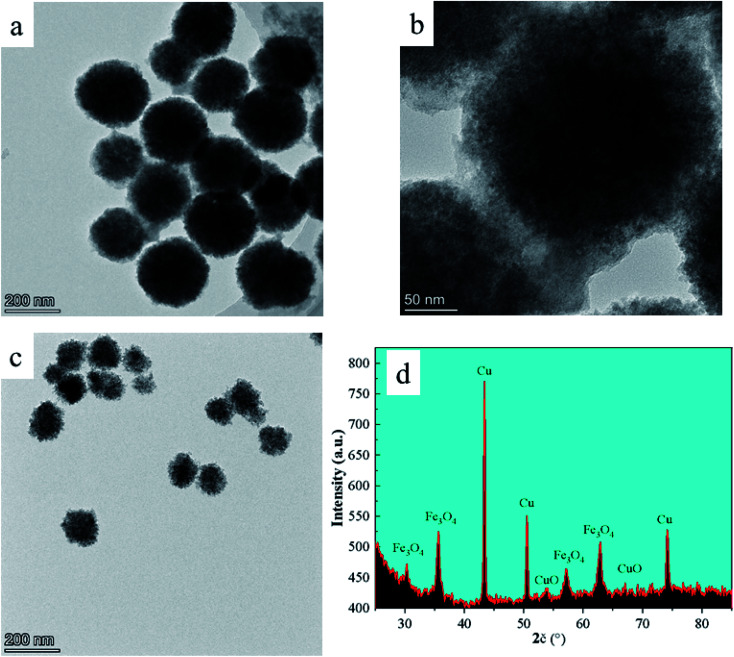
TEM images (a–c) and XRD pattern (d) of FC nanocomposite.

TEM was used to describe the morphology and size of the produced material, and the findings are displayed in Fig. 1a–c. The generated FC has a monodisperse spherical shape with a particle size of 230 ± 20 nm and a rough surface, as shown in the picture. The primary explanation for this is that copper chloride and ferric chloride were progressively converted to FC nuclei at elevated temperatures using ethylene glycol and sodium citrate. The FC nuclei progressively gathered and developed into a spherical shape with a rough surface as the reaction continued. Powder X-ray diffraction was used to further investigate the crystal structure of the FC (XRD). The findings are depicted in Fig. 1d. The diffraction peaks at 30.28, 35.85, 57.30 and 62.57° correspond to the Fe_3_O_4_ spinel structure (220), (311), (511) and (440) planes^27^ (standard diffraction peak card JCPDS 19-0629). The diffraction peaks at 53.48 and 67.9 nm correspond to the (020) and (113) planes of the CuO monoclinic phase, respectively (standard diffraction peak card JCPDS 05-0661). The diffraction peaks at 43.30, 50.43 and 74.13° of 2*θ* values were assigned to the cubic phase Cu crystallographic planes (111), (200) and (220).^28^ FC included spinel Fe_3_O_4_, monoclinic phase CuO, and cubic phase Cu, as determined by XRD.

### 3.2 Elemental analysis

**Fig. 2:**
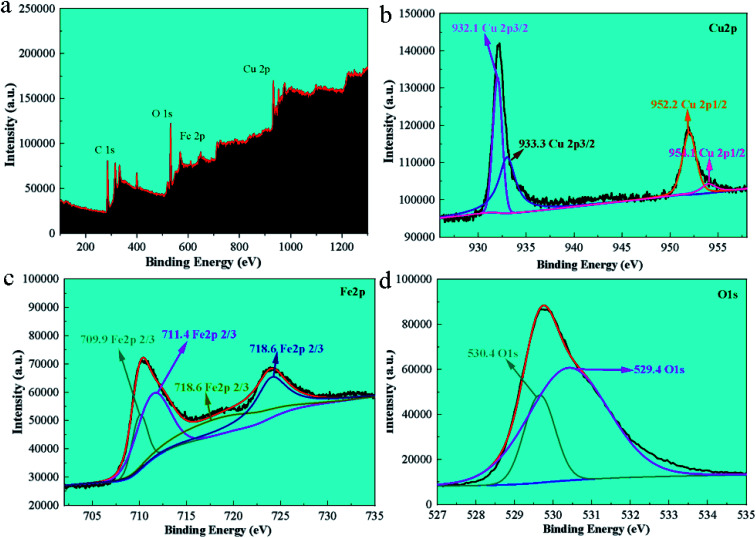
The XPS survey spectrum of FC and high resolution of Cu 2p (b), Fe 2p (c), and O 1s (d).

The elemental composition and valence state of the material were determined by XPS, as seen in Fig. 2a, which correspond to the binding energy spectrum peaks of single Cu, Fe, and O elements, respectively. The peaks at 932.8 eV and 952.7 eV correspond to the binding energy of Cu 2p in the material,^27^ whereas the peaks at 711.2 eV and 725.0 eV correspond to the binding energy of Fe 2p (Fig. 2b and c). In Fig. 2b, two peaks at 951.8 and 931.8 eV correspond to the binding energies of Cu 2p_1/2_ and 2p_3/2_, respectively, suggesting that the produced catalyst existed in the form of Cu^2+^. The peaks at 530.8 eV and 529.5 eV are the binding energies of O 1s (Fig. 2d).

Additionally, a peak at 933.5 eV was found, confirming the presence of elemental Cu in the catalyst. Fe 2p centering peaks at 711.1 eV (2p_3/2_) and 724.2 eV (2p_1/2_) are seen in Fig. 2b, confirming the existence of Fe on the surface of FC as Fe^2+^ and Fe^3+^ ions.^29^ The spectral peaks 530.8 and 529.5 eV in O 1s match the O^2−^ element in CuO and Fe_3_O_4_,^28^ indicating that the Fe_3_O_4_/CuO composites included Cu^2+^, Cu, O^2−^, Fe^2+^ and Fe^3+^.

### 3.3 Ultraviolet and magnetic analysis

**Fig. 3:**
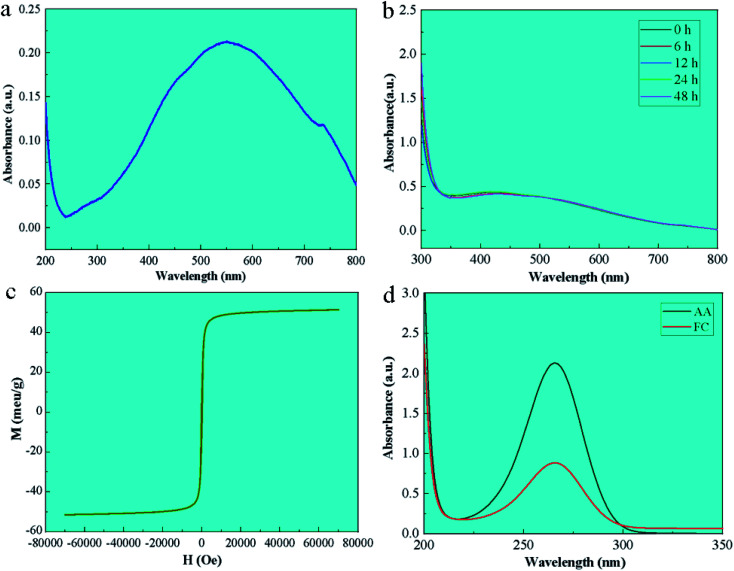
The UV-vis absorption spectrum of FC (a), the stability test of FC (b), the saturation magnetization of FC (c), and UV-vis diffuse reflectance spectra of FC and FAN samples produce ROS against the degradation peak of ascorbic acid (d).

The UV-vis absorption spectrum of FC is shown in Fig. 3a. As seen, FC has a distinct absorption peak at 547 nm, while Fe_3_O_4_ has no absorption peak in the visible light spectrum,^19^ but elemental copper exhibits a distinct absorption peak between 500 and 550 nm,^17^ confirming the existence of copper in FC.

The stability test results of FC in phosphate buffer was shown in Fig. 3b. The UV-vis result shows that the changes in the position and area of the UV absorption peaks of FC at 0 h, 6 h, 12 h, 24 h and 48 h are not obvious, and no new absorption peaks appear, which proves that FC is relatively stable in phosphate buffer.

The magnetic characteristics of FC at room temperature were determined using a vibrating sample magnetometer (VSM) in the range of −10 000 Oe to 10 000 Oe. The VSM results reveal that FC has high magnetic characteristics. Magnetic characteristics are reflected in the image by the hysteresis loops. Magnetic saturation (MS) of 51.56 emu g^−1^ is associated with FC.

### 3.4 ROS monitoring

FC contains Cu^2+^, Cu^0^, Fe^2+^ and Fe^3+^ ions, with *E*_(Cu^+^/Cu)_ = 0.52 V, *E*_(Cu^2+^/Cu^+^)_ = 0.159 V, *E*_(Fe^3+^/Fe^2+^)_ = 0.77 V. Cu^0^, being unstable in the air, is apt to change into Cu^+^, which further disputes itself to generate Cu^2+^ and Cu. Fe^3+^ can generate Fe^2+^ and Cu^2+^ when it is combined with Cu^0^, and Fe^2+^ generates Fe^3+^ and other free radical compounds when combined with oxygen in the air, releasing ROS in such a process of electron circulation and migration to give the material bacteriostasis effect and to promote plant growth in collaboration with Cu^2+^.

Ascorbic acid can be oxidized by ROS to deoxy ascorbic acid.^19^ To further elucidate the antibacterial mechanism of FC, ascorbic acid with UV-visible absorption peaks was employed to measure the rate of ROS formation (shown in Fig. 3c). The ROS study revealed that the material FC produces ROS in the medium after 20 minutes, indicating the presence of electron migration and ROS formation in FC. After 20 minutes of culture, the absorption peak at 266 nm corresponds to the typical absorption peak of ascorbic acid, and the degradation rate of FC toward ascorbic acid is 32.7%.

### 3.5 Antibacterial studies

**Fig. 4:**
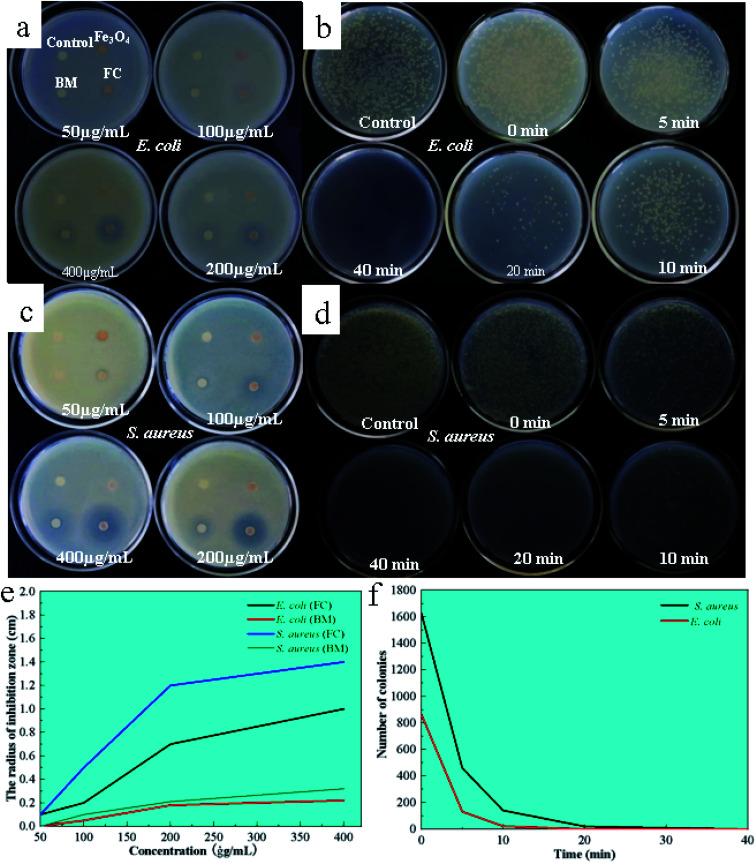
Inhibition zones of FC, Bordeaux mixture (BM), Fe_3_O_4_ against *E. coli* (a) and *S. aureus* (b); image of LB agar culture plate inoculated *E. coli* (c), and *S. aureus* (d) treated in a short time while with FAN (200 μg mL^−1^); the results correspond to the data analysis is (e) and (f).

The antibacterial activity of the FC was investigated using the filter paper diffusion technique against model bacteria *E. coli* and *S. aureus*, with Bordeaux mixture and ferrosoferric oxide serving as controls. The findings are depicted in Fig. 4. As shown in Fig. 4a, there was no antibacterial circle around the filter paper when the Fe_3_O_4_ concentration was between 50–400 μg mL^−1^, indicating that Fe_3_O_4_ has no antibacterial activity in this range. On the other hand, the Bordeaux mixture exhibits antibacterial activity against *S. aureus* at a 200 μg mL^−1^ concentration and *E. coli* at the concentration of 400 μg mL^−1^, showing that the Bordeaux mixture has a greater antimicrobial efficacy against *S. aureus* (Fig. 4c).

At 200 μg mL^−1^, FC demonstrates antibacterial activity against *E. coli* and *S. aureus*, as shown by clear antibacterial circles around the respective filter sheets, with bigger circles for *S. aureus*. FC had a 40.5-fold antibacterial activity against *E. coli* at the same concentration while having a 60.5-fold antibacterial efficacy against *S. aureus*. By contrast, the present work observed that FC has superior antibacterial effectiveness at 200 μg mL^−1^, which was four times higher than the Bordeaux combination.

### 3.6 Time measurement of antibacterial efficiency

To further investigate the antimicrobial efficacy, its inhibition was examined by colony counting at concentrations of 200 μg mL^−1^ for 0, 5, 10, 20 and 40 min. The findings and analysis of the data are depicted in Fig. 4b and d. In Fig. 4b and d, the reference (water) contained 1982 *E. coli* and 1924 *S. aureus* colonies. The number of colonies reduced as time passed, with no *E. coli* colonies forming after 40 minutes, indicating that the FC was entirely antibacterial for *E. coli* within 40 minutes at a 200 μg mL^−1^ concentration. Within 20 minutes, no *S. aureus* colonies were detected, showing that the FC was utterly bactericidal to *S. aureus*.

### 3.7 Microcalorimetric analysis of bacterial growth

**Fig. 5:**
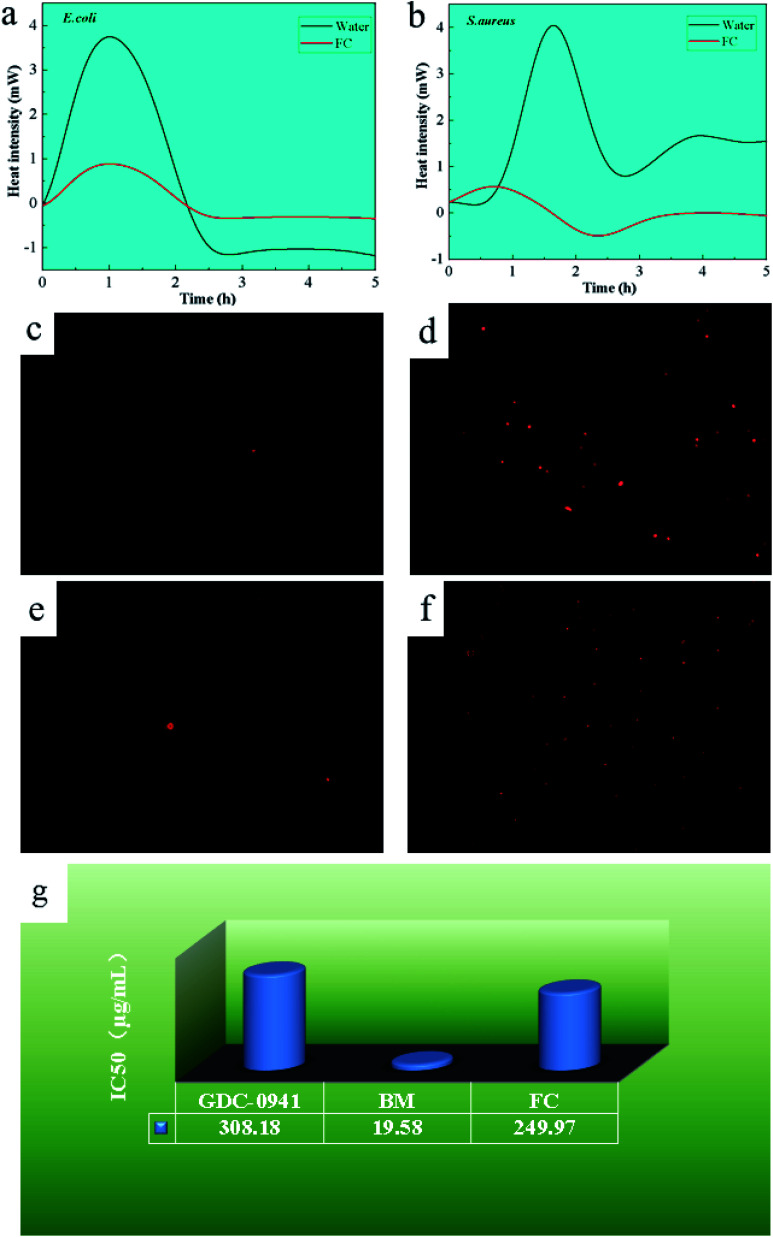
Microcaloric analysis of FAN on *E. coli* (a) and *S. aureus* (b); PI staining analysis of FAN on *E. coli* (c and d) and *S. aureus* (e and f). Viability of MCF-7 cells exposed to GDC-0941, BM and FAN nanoparticles (g).

Minimal inhibitory activity was determined by monitoring changes in heat generated by several stages of bacterial metabolism using microcalorimetric analysis techniques. Bacterial survival was estimated by measuring the difference in heat generation throughout the adjustment, log, stationary and decline phases. Fig. 5a and b illustrated the results of the microcalorimetric analysis with a material concentration of 200 μg mL^−1^. After introducing the material to *E. coli* and *S. aureus*, the adaptation period and exponential period were delayed, with *E. coli* having a shorter adaptation time. The heat flux created by the procedure is approximately 1/5 that of untreated bacteria in the case of *E. coli* and 1/10 in the case of *S. aureus*.

This can be attributed to the bacterial resistance mechanism. As trace elements in microorganisms,^11,13,20^ Cu and Fe demonstrated early adaption and self-resistance and later inhibition when the external concentration exceeded their minimal inhibitory concentration. The material inhibits *S. aureus* more effectively than *E. coli* because *S. aureus* cell walls have a high proportion of phospholipid bilayers and a low proportion of protein.^22,27,29^ Due to the fact that bacterial resistance mechanisms depend on the expression of resistance genes in plasmid DNA proteins, the more protein in the cell wall, the more probable that resistance gene expression is activated.^29^ As a result, *S. aureus* is more susceptible to the FC.

### 3.8 PI staining analysis

PI may selectively bind DNA and RNA as a fluorescent dye in bacteria. When bacteria die, PI penetrates the bacterium and acts on nucleic acid, generating a red spot-like material that may be seen under an inverted fluorescence microscope, as seen in Fig. 5c–f. The presence of red spot-like compounds was lower in the control group of *E. coli* and *S. aureus*, which can be related to the natural aging process of bacteria. With the addition of the FC, more red spots were noticed in *E. coli* and *S. aureus*. Furthermore, as compared to *E. coli*, more red spots were found in *S. aureus*, demonstrating the material better antibacterial activity against *S. aureus*, consistent with the above analytical results and previous reports.^30–34^

With the addition of the FC, more red spots were noticed in *E. coli* and *S. aureus*. Furthermore, as compared to *E. coli*, found more red spots in *S. aureus*, demonstrating the material better antibacterial activity against *S. aureus*, consistent with earlier analytical results.

### 3.9 Cytotoxic analysis

To investigate the material biocompatibility, the half-maximal inhibitory concentration (IC_50_) of Pictilisib (GDC-0941) has been used, a commercially available antitumor agent, as a reference, and human breast epithelial cells as simulation cells to investigate the material's half maximal inhibitory concentration, as shown in Fig. 5g. The IC_50_ of GDC-0941 was determined to be 308.18 μg mL^−1^. The IC_50_ values for the Bordeaux combination and the FC were 19.58 μg mL^−1^ and 249.97 μg mL^−1^, respectively. The results show that the toxicity level of the FC was 1.23 times higher than that of the agent GDC-0941 but significantly lower than that of the Bordeaux combination, representing 7.83% of the Bordeaux mixture's toxicity level. The explanation for this might be that Fe, as a trace element in eukaryotic cells, is less poisonous than Cu. As a result, FC is less hazardous.^35–38^

### 3.10 Effects on mung bean germination

**Fig. 6:**
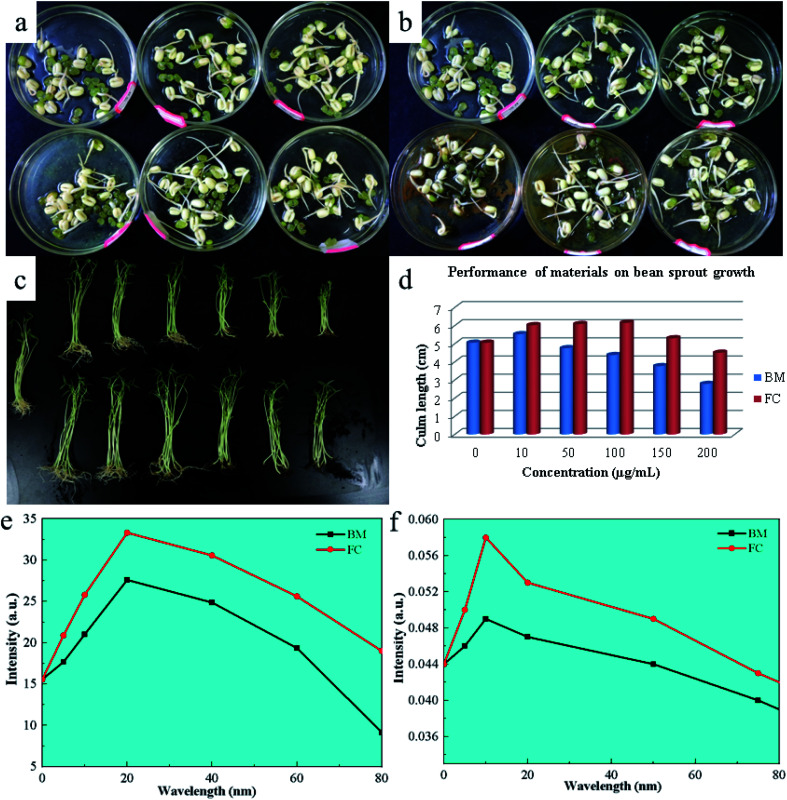
Photographs of the results of BM and FAN on mung bean sprouting (a and b); photographs of the results of the effect of materials on root activity and chlorophyll content in the growth of mung bean (c), also corresponding data analysis results (d–f).

Mung bean exhibits anaerobic respiration during the early stages of germination, with its seed skin relaxing and expanding after water absorption, allowing oxygen to enter the seed and promote aerobic respiration. During this step, metabolism speeds up, and more nutrients are required to hasten seed germination. Fig. 6 depicts the outcomes of using FC in mung bean germination (a and b). The concentration of the Bordeaux combination (tested at 10, 50, 100 and 200 μg mL^−1^) increased root elongation and thickness. Nonetheless, at a 400 g mL^−1^ dosage, they fell dramatically. When applied to mung bean seeds, FC revealed comparable capabilities in promoting germination, as well as an inhibitory impact on seed germination at a dosage of 400 μg mL^−1^. Still, the effect was smaller than that of the Bordeaux combination. Fig. 6c depicts the material impact on physiological indicators of mung bean seedlings. Capillary roots are more active and stems and leaves grow thicker when the concentration of the Bordeaux combination is 10 μg mL^−1^ and 50 μg mL^−1^, as shown in the picture. However, when the concentration is between 100 and 400 μg mL^−1^, it demonstrates increasing inhibitory effects as the concentration increases.

The experiment on the root activity and chlorophyll content of mung bean seedlings shows that FC can accelerate the development of mung bean capillary roots and increase chlorophyll content. Meanwhile, at concentrations ranging from 10–100 μg mL^−1^, FC might promote root and leaf development in mung bean seedlings. Nonetheless, it had similar inhibitory effects on seedlings at concentrations ranging from 200 to 400 μg mL^−1^, but was less toxic than the Bordeaux combination at the same dose. Fig. 6d–f depict the appropriate data.

### 3.11 Antibacterial and growth-promoting mechanism of FC

**Fig. 7:**
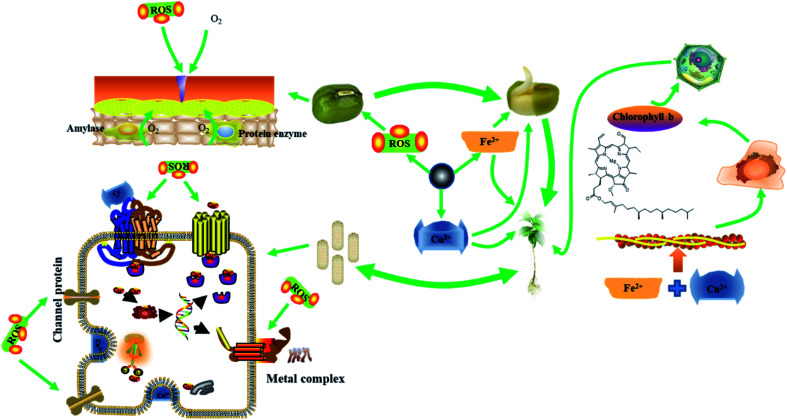
The possible mechanisms of antibacterial and growth-promoting by FC.

The possible mechanisms of antibacterial and growth-promoting by FC are shown in Fig. 7. As crucial trace elements in plant development, Cu ions and Fe in FC perform critical roles in electron transport and the creation of main functional proteases in plant metabolism. To enhance plant development, Fe and Cu ions can speed up the production of superoxide dismutase, ascorbate oxidase, and chlorophyll.^2,7,11–13^

Because of the wrapping of the seed coat, seeds are in anaerobic respiration during the early stages of germination. When FC is administered at a concentration less than 10 g mL^−1^, it reacts with medium water to form ROS to oxidize the seed coat, causing the seed coat to rupture and allowing the seed to start the aerobic respiration process, hence speeding seed germination. As a result, mung bean sprouts get thicker.^15,20,39^

During the growth stage, Fe^2+^, Fe^3+^ and Cu^2+^ released by FC are electrostatically absorbed in the plant cell wall and reduced by reductase into Fe^2+^ and Cu^+^, which enter the cell *via* ion channel proteins and are transported to chloroplasts, mitochondria, vacuoles, and other organelles in collaboration with chaperone proteins.^5,7,12,18,39^

In plants, Fe is primarily involved in electron transport. Fe increases plant metabolism and acts synergistically with Cu to accelerate the development of mung bean roots and enhance the level of green pigment when administered to mung bean sprouts at an adequate concentration.^39,40^

Excess Cu^+^, on the other hand, can be harmful if it is loose in the cytoplasm. As a result, significant levels of Cu^+^ are retained in the vacuole, cytoplasm, or cell wall. Mung bean roots can store a high quantity of Cu^+^ and convey it to terrestrial tissues and organs *via* transfer proteins.^4,7,11,13^ As a result, the roots are more susceptible to FC concentrations. Mung bean has different absorption efficiency of Fe and Cu at different stages, and the study of alternating Fe and Cu is a meaningful study for the growth of mung bean at each stage, which will be explored in further research.

Bacteria have a lower need for Cu than plants, therefore Cu ions at a particular concentration can boost plant development and cause harm to bacteria.^8,10,39,41^ Because the bacterial surface has a high concentration of phenolic hydroxyl and carboxyl groups, the surface is retained in an acidic environment, where FC performs the Fenton reaction, producing additional ROS to boost antibacterial activity.

When too much Cu^2+^ penetrates the bacterial wall, it attaches to the iron–sulfur cluster proteins in the cell membrane, causing secondary structural degeneration. When the FC concentration is less than 100 μg mL^−1^, it may not only kill bacteria but also stimulate mung bean seed germination, root activity, and chlorophyll content in mung bean sprouts.

Furthermore, the components of FC are mainly Fe^2+^, Fe^3+^, Cu^0^ and Cu^2+^ with strong electron migration rate and antibacterial activity, and because it contains Fe and Cu elements, it can have a certain promotion effect on plant growth at moderate concentrations, and plants have different requirements for Fe and Cu elements during the growth process, and the application of different elements at the growth stage for the application of different elements at the growth stage has important research and practical value for plant growth, which we will explore in further studies. In addition, if Fe_3_O_4_ is used as the nucleus and Cu^0^ and Cu^2+^ or Cu^2+^ and Cu^0^ are loaded on the surface in turn due to Fenton reaction and interfacial effect can produce a certain amount of ROS,^42–44^ theoretically, it can have some antibacterial activity because Cu^0^ and Cu^2+^ have different redox potentials and can spontaneously carry out redox reactions to produce more free electrons and Cu intermediates and cause more damage to bacteria, so it is important for bacterial inhibition research and application. However, this material needs to be synthesized by layer assembly, multi-step reaction, and exhaustive characterization and antibacterial activity testing, while the present material is a composite of four elements, Fe^2+^, Fe^3+^, Cu^0^ and Cu^2+^. Therefore, with Fe_3_O_4_ as the core, loading Cu^0^ and Cu^2+^ or Cu^2+^ and Cu^0^ on the surface in turn will require further study.

## 4. Conclusion

The antibacterial efficiency of the FC against *E. coli* and *S. aureus* was 100% within 20 minutes at a concentration of 100 μg mL^−1^, which is four times greater than that of the Bordeaux mixture at the same concentration, with better antimicrobial activity against *S. aureus*. The antibacterial investigations revealed that the FC could kill germs and severely destroy bacterial cell walls. The material toxicity to mammalian cells was 1/8 that of the Bordeaux combination. Mung bean seeds may also benefit from the material's germination and development. With toxicity of 1/8 that of the Bordeaux combination, the FC has the potential to be more useful in agricultural planting.

## Conflicts of interest

The authors declare that they have no known competing financial interests or personal relationships that could have appeared to influence the work reported in this paper.

## Supplementary Material
